# Increasing access to the MDR-TB surveillance programme through a collaborative model in western Kenya*

**DOI:** 10.1111/j.1365-3156.2011.02933.x

**Published:** 2012-03

**Authors:** Paul H Park, Cornelius Magut, Adrian Gardner, Dennis O O'yiengo, Lydia Kamle, Bernard K Langat, Nathan G Buziba, E Jane Carter

**Affiliations:** 1 Duke University Medical CenterDurham, NC, USA; 2 Moi University School of MedicineEldoret, Kenya; 3 Warren Alpert Medical School of Brown University, ProvidenceRI, USA; 4 Division of Leprosy, Tuberculosis, and Lung DiseaseNairobi, Kenya

**Keywords:** tuberculosis, multidrug-resistant, microbial sensitivity tests, Kenya, delivery of health care

## Abstract

**Objective:**

Kenya, like many resource-constrained countries, has a single mycobacterial laboratory, centrally located in Nairobi, with capacity for drug-susceptibility testing (DST) – the gold standard in diagnosing drug-resistant tuberculosis. We describe and evaluate a novel operational design that attempts to overcome diagnostic delivery barriers.

**Methods:**

Review of the public DST programme identified several barriers limiting access: lack of programme awareness amongst physicians, limited supplies, unreliable transport and no specimen tracking methods. Staff visited 19 clinic sites in western Kenya and trained healthcare providers in regard to the novel diagnostics model. Provincial laboratory registries were reviewed to assess utilization of DST services prior to and after programme modification.

**Results:**

Onsite training consisted of the inclusion criteria for re-treatment patients – the high-priority group for DST. Additionally, infrastructural support established a stable supply chain. An existing transport system was adapted to deliver sputum specimens. Task shifting created an accession and tracking system of specimens. During the 24 months post-implementation, the number of re-treatment specimens from the catchment area increased from 9.1 to 23.5 specimens per month. In comparing annual data pre- and post-implementation, the proportion of re-treatment cases receiving DST increased from 24.7% (*n* = 403) to 32.5% (*n* = 574) (*P* < 0.001), and the number of multidrug-resistant (MDR) TB cases increased from 5 to 10 cases.

**Conclusion:**

The delivery model significantly increased the proportion of re-treatment cases receiving DST. Barriers to accessing the national MDR-TB surveillance programme can be overcome through an operational model based on pragmatic use of existing services from multiple partners.

## Introduction

A previous history of tuberculosis (TB) treatment has been identified in a number of epidemiologic studies as a risk factor for drug-resistant tuberculosis and multidrug-resistant TB (MDR-TB) (Espinal *et al.* 2001; Faustini *et al.* 2006). The need for ‘re-treatment’ may reflect undiagnosed primary drug-resistant TB, poor adherence or defaulting, leading to acquired resistance, short-term relapse of TB or re-infection. Therefore, re-treatment patients are a high-priority group for whom culture and drug-susceptibility testing (DST), the gold standard for diagnosing MDR-TB, should be performed.

According to a 2010 WHO report, only 18% of re-treatment tuberculosis cases in Kenya received DST despite national guidelines and programmatic support for this practice (World Health Organization 2010b). Kenya currently has only one laboratory, the Central Reference Laboratory (CRL) in Nairobi, with DST capacity. The current national TB re-treatment surveillance programme as established by the Division of Leprosy, TB, and Lung Disease (DLTLD) employs countrywide specimen transport routing systems. The DLTLD contracts a private courier company to ship all sputum specimens of ‘target recipient patients’ to the CRL for culture and DST at no financial cost to the patient. TB clinic staff at the local level identifies these patients, collects sputa and delivers specimens to the courier company's drop point. Samples are transported to the CRL on at least a weekly basis. Paper results are returned to the clinics in the same manner.

This centralized approach has been used in other settings with limited laboratory services but frequently leads to delays in turnaround time up to 5 months (Yagui *et al.* 2006; Shin *et al.* 2008; Singla *et al.* 2009). During this delay, patients with drug-resistant TB may continue failing empirical regimens, leading to community transmission and resistance amplification (Farmer *et al.* 1998; Furin *et al.* 2000). Although successful treatment of MDR-TB has been demonstrated in resource-poor settings (Mitnick *et al.* 2003; Van Deun *et al.* 2010), studies have shown mortality rates of patients awaiting DST results and ultimately diagnosed with MDR-TB to range from 12 to 50% (Singla *et al.* 2009). Harries *et al.* (2004) published a report from Malawi showing that despite implementation of a new bus transport system for re-treatment specimens, 60% of cases resulted in a sample not being received by the central laboratory. Beyond transportation, a variety of other operational challenges related to rural clinics leads to delay, including frequent staff turnover with inadequate training regarding the re-treatment programme and stock-outs of sputum collection bottles and laboratory forms (Harries *et al.* 2004; Shin *et al.* 2008; Griffin *et al.* 2009; Singla *et al.* 2009). Clinical staff is generally not trained to set up accession and tracking systems in the same manner that laboratories are; many clinics have no record of which patient had a specimen collected or when the specimen was sent to the laboratory. Logistical dilemmas in the operational mechanism may result in up to 29% of confirmed MDR-TB cases having ‘lost’ results (Griffin *et al.* 2009). Consequently, screening of high-risk patients is not completed, and health system-based problems cannot be identified because of the lack of documentation.

Decentralization of MDR-TB surveillance services may occur through the addition of regional laboratory centres with DST capacity. However, such an approach is too costly for many of the high-burden TB countries. The recent development of Xpert® Mycobacterium tuberculosis/rifampicin and other cartridge-based, rapid diagnostic systems will be useful for point of care diagnosis of TB and drug-resistant TB; however, these diagnostic tools do not eliminate the need to continue to invest in culture and DST capacity (World Health Organization 2011). DST decentralization or implementation of rapid testing methods will only minimally impact patient outcomes if the pre- and post-DST logistical barriers are not addressed (Shin *et al.* 2008).

Here, we describe a healthcare delivery model that increases accessibility to DST services in western Kenya by addressing operational barriers. This modification of the national TB re-treatment surveillance programme focuses on establishing partnerships to collaboratively build upon existing infrastructure.

## Methods

Moi University School of Medicine (MUSOM) and Moi Teaching and Referral Hospital (MTRH) are located in Eldoret, 300 km west of Nairobi. MTRH is one of two established referral hospitals in Kenya, supplying both local primary care and secondary referral care (North Rift Valley and Western Provinces). In 2000, MUSOM and MTRH, with their U.S. partners, established AMPATH, originally titled Academic Model for Prevention and Treatment of HIV/AIDS and now the Academic Model Providing Access to Healthcare (AMPATH). Academic Model Providing Access to Healthcare consists of 23 HIV care clinics incorporated into the Ministry of Health units with a centralized AMPATH Reference Laboratory (ARL) and a transport system for personnel and laboratory specimens. The ARL includes the Mycobacteriology Reference Laboratory (MRL), established under a Foundation for Innovative New Diagnostics (FIND) grant. The MRL is capable of smear, liquid and solid culture, and DST; however, funding to support routine culture and DST has not yet been established.

The CRL routinely requires 12 weeks or more for completing DST because of a multistep process that includes use of solid media. Isolation of mycobacteria on solid media can take up to 8 weeks. Thereafter, DST is performed with Mycobacteria Growth Indicator Tube (MGIT), which involves a liquid medium.

In October 2007, MRL staff visited 19 health facilities collaborating with the AMPATH programme. With the support of the DLTLD, MRL staff provided training to all physicians, nurses and laboratory technicians of the public chest and HIV clinics as well as district Ministry of Health coordinators regarding the inclusion criteria for ‘re-treatment cases’ and their significance. Training also focused on accessing the AMPATH transport and laboratory support systems to facilitate transporting of specimens, stocking of sputum collection bottles and tracking of laboratory results.

A pre- and post-programme retrospective evaluation was performed. All re-treatment cases documented by the MRL and CRL were included in the study. Amongst the documented re-treatment cases, no cases were excluded. MRL tracking database was reviewed to assess utilization of the services and results turnaround times. The national CRL registry provided provincial-level data on the proportion of re-treatment cases receiving DST and MDR-TB diagnosis. Fisher's exact test was used to assess for statistical significance.

## Results

### Diagnostics delivery model

**Figure 1:**
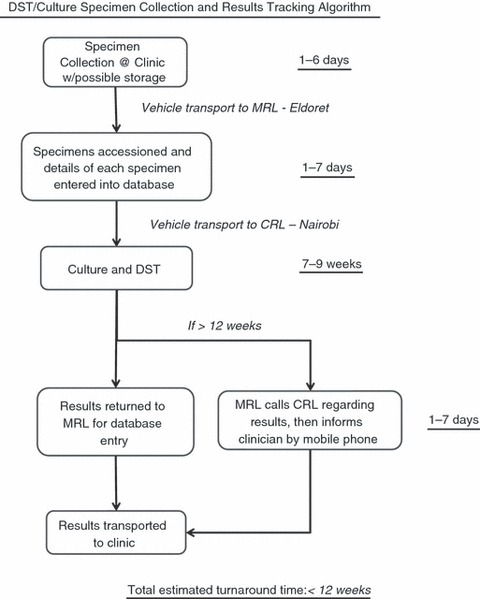
Algorithm for DST/culture specimen collection, accession and electronic results tracking as implemented at 19 clinics in the catchment area of the MRL in western, Kenya.

Upon completion of on-site training at 19 satellite health facilities, a novel logistical model was in place for accessing the national MDR-TB surveillance programme (Figure 1). TB care providers send all re-treatment specimens to the MRL via AMPATH vehicles. AMPATH vehicles routinely visit these health facilities 1–4 times per week, carrying supplies, specimens and personnel. TB care providers were asked to schedule sputum collection of re-treatment cases on or immediately prior to the day of transport delivery whenever possible. For those specimens requiring storage, sputum-containing bottles were stored in a designated site devoid of sunlight and excess heat. Upon arrival to the MRL, laboratory technicians accession specimens and electronically document all relevant details: patient identification, health facility, ordering physician, date of specimen arrival, date of shipment to CRL, date of results arrival and culture/DST results.

All specimens tracked through the programme are appropriately stamped by MRL staff, so the CRL can provide results to the treatment clinics via the MRL. Upon receipt of results from the CRL, the MRL staff logs the results in the electronic database. Paper laboratory results are then returned to the ordering clinic via AMPATH vehicles. MRL staff additionally report any drug resistance to the requesting clinician by mobile phone.

The electronic database allows for establishment of a steady supply chain in addition to the *re-tracking policy* (Figure 1). Quarterly, MRL staff review the number of specimens received per site and deliver an equivalent number of culture bottles, specimen bags and request forms to the clinic. An inventory of all specimen collection supplies was documented during the initial site visits, and MRL staff provided an additional surplus on that day as needed. The *re-tracking policy* is an algorithm for identifying delayed or potentially lost results. Specimen tracking in the electronic database is performed bimonthly. Per protocol, if the MRL does not receive a specimen report from the CRL within 12 weeks, MRL staff contacts the CRL by mobile phone to ascertain the status of the specimen. Results are then either identified, pending, deemed as inadequate sample (cracked bottle or contaminant) or ‘lost’.

### Post-implementation quantitative outcomes

During the 24-month period after the re-treatment surveillance programme was instituted, the MRL facilitated an average of 23.5 specimens per month from the catchment area, *vs*. 9.1 specimens per month before the programme started (Table 1). The proportion of re-treatment cases from the catchment area receiving DST annually increased from 24.7% (*n* = 403) to 32.5% (*n* = 574) between 2006 and 2008 (*P* < 0.001). The number of MDR-TB cases identified in North Rift Valley and Western Provinces also increased from five cases in 2006 to 10 cases in 2008. The number of participating satellite clinics rose to >25 after the district DLTLD staff elected to expand the programme to additional sites. Sputum samples from neighbouring satellite clinics are now dropped off at AMPATH-connected sites on the day of sputum transport.

**Table 1:** Pre- and post-programme values in relation to implementation of decentralization model that occurred in the 4th quarter of 2007

	Pre-implementation	Post-implementation
No. specimens collected at MRL	9.1	23.5
No. re-treatment cases in catchment area	1629	1765
% Re-treatment cases completing DST in catchment area	24.7	32.5*
No. of MDR-TB cases identified in catchment area	5	10
Turnaround time (weeks) of MRL-accessioned specimens	N/A	11.7

* *P* < 0.001.

The *re-tracking policy* was used for 33% (*n* = 206) of the specimens, and 63% (*n* = 130) of re-tracked specimens were successfully recovered to produce a documented result. Therefore, 12% (*n* = 76) of all re-treatment cases at the MRL were deemed as ‘lost’. The average turnaround time for specimen results was 11.7 weeks including those that required the re-tracking policy; average turnaround time for specimens not requiring the re-tracking policy was 9.6 weeks.

## Discussion

The modified TB retreatment surveillance programme at AMPATH MRL resulted in a larger proportion of re-treatment patients accessing DST service within the catchment area. The programme operated through collaborations with various stakeholders that resulted in a series of pragmatic operational interventions: education of staff regarding the programme guidelines, establishment of supply chain for laboratory forms and bottles, utilization of an existing transport system and a dedicated tracking system for results.

While baseline data are not available for turnaround time, the MRL achieved its target goal of <12 weeks. We also note that more than half of the delayed specimens that would potentially be considered ‘lost’ were identified to produce a reportable result. The need to use the *re-tracking policy* for nearly one-third of all samples is a reflection of the reality of the situation – limited organizational infrastructure and inconsistent work flow. This points to the need for a standardized accession and tracking system which the previous clinic-based system did not have.

The diagnostics delivery system was accepted by the local clinics as reflected by the DLTLD's decision to expand the programmatic structure to include more rural sites. Based on informal interviews with local healthcare providers, the acceptance of the programme has been due to the increased reliability of return of results, improved turnaround time, frequency of deliveries, stable supply chain and more proximal drop points for specimen delivery.

Two major challenges to both the uptake and sustainability of the re-treatment surveillance programme are maintenance of medical staff knowledge and maintenance of the culture bottles supply chain. MRL staff revisited three sites where participation rates were low. These site visits consistently demonstrated high staff turnover with new staff having limited knowledge of the programme protocols. The lack of adherence by clinicians to re-treatment protocols can have a devastating impact on the ability to identify MDR-TB cases in the community. In one study from Peru, perfect adherence by clinical staff to national re-treatment guidelines would have resulted in a 50-fold increase in DST requests (Griffin *et al.* 2009). Regarding the supply chain, intermittent national shortages of culture bottles inhibited the MRL from dispensing supplies in a timely manner. Presently, a monthly re-treatment surveillance report is reviewed by the central team to target sites where re-education or examination of supply chain issues is warranted. Formal agreements between laboratories regarding prompt delivery schedules of supplies are needed. Lastly, utilization of internet-based medical records would also facilitate results tracking and reduce turnaround time (Shin *et al.* 2008).

Limitations of the study include the inability to identify the independent impact of each element of the delivery model (training, supply chain, transport and re-tracking). A pre- and post-test of all healthcare personnel involved in a training session would be of value. In addition, further operational research comparing intervention clinics to non-intervention clinics with baseline data would provide stronger controls accounting for potential confounding variables, including geographic temporal trends. Lastly, even after implementation of this protocol, there still remains much room for improvement regarding diagnostic delays and reliability of results.

We were unable to calculate the cost of task shifting by laboratory staff and transportation. However, we should note that no new job positions or transportation routes were created for the implementation of this modified national re-treatment programme. The only new items were the laboratory cell phone and cell phone airtime. The major lessons of our programme are twofold: (i) identification of existing health system resources – the expertise of the regional laboratory staff in training and access, the AMPATH transport system and the DLTLD's supply chain with the MRL's inventory monitoring – which can be co-opted to assist national- or provincial-level programmes; (ii) the establishment of a tracking system that allows for programme monitoring and evaluation. According to a 2006 WHO report, only 9% of re-treatment tuberculosis cases received DST in the WHO African region with a goal of 90% by 2015 (World Health Organization 2006, 2010a, 2010b). This pragmatic approach to examine coexisting complementary programmes and to establish monitoring systems may be a feasible and immediate approach to increasing this figure.

Harries *et al.* (2004) concluded that while the new transport system alone was seen as partially beneficial, there exists a need for further operational research into the details of systematically collecting and transporting specimens without becoming ‘lost’. The advent of novel diagnostics such as Xpert® (Boehme *et al.* 2010) has also acknowledged the inevitable barriers of decentralization to rural clinics related to ‘supply chain management, reagent storage and calibration’ (Boehme *et al.* 2011). Unless resources are decentralized to every dispensary, transport systems that tie diagnostics to point of care will continue to be needed. Clear-eyed programme analysis and a practical operational plan that unites available services and establishes monitoring programmes does not require large investments and can improve results as seen in our programme.
